# Laboratory evaluation of anti-plaque and remineralization efficacy of sugarless probiotic jelly candy supplemented with natural nano prebiotic additive

**DOI:** 10.1038/s41598-023-37645-5

**Published:** 2023-07-06

**Authors:** Hanaa M. Elgamily, Samah M. El-Sayed, Hoda S. El-Sayed, Ahmed M. Youssef

**Affiliations:** 1 Restorative and Dental Materials Department, Oral and Dental Research Institutes, National Research Centre, 33 El Bohouth St., Dokki, Giza, 12622 Egypt; 2 Dairy Department, Food Industries and Nutrition Research Institute, National Research Centre, 33 El Bohouth St., Dokki, Giza, 12622 Egypt; 3 Packaging Materials Department, National Research Centre, 33 El Bohouth St., Dokki, Giza, 12622 Egypt

**Keywords:** Microbiology, Health care

## Abstract

We evaluated the anti-cariogenic effect of an experimental synbiotic compound containing probiotic *Lacticaseibacillus rhamnosus* (NRRL B-442)-based jelly candy supplemented with natural prebiotic grape seed extract (GSE) in a nanoemulsion formula on the colonization and establishment of *Streptococcus mutans* (ATCC 25175) and *Actinomyces viscosus* (ATTCC 19246) biofilms through counting colony forming units, scanning electron microscopy (SEM), and transmission electron microscopy (TEM). We were then analysing the remineralizing effect of synbiotic jelly candy on human enamel surface lesions using Vickers microhardness testers, atomic force microscopy (AFM), SEM, energy-dispersive X-ray spectroscopy (EDAX), and confocal laser scanning microscopy (CLSM) at three stages (sound, after demineralization, and after pH cycling). We found after 21 days of treatment of the pH-cycled enamel discs with jelly candy for 10 min twice daily, a 68% decrease in *S. mutans* colony formation, reducing biofilm development, trapping *S. mutans* visualized in jelly candy under SEM examination, and significantly altering the morphological structure of these bacteria under TEM analysis. For remineralization measurements, statistically significant differences in microhardness integrated mineral loss, and lesion depth through CLSM between demineralization and treatment stages. These findings provide an effective anti-cariogenic synbiotic compound of grape seed extract and probiotic jelly candy with potential remineralizing activity.

## Introduction

Dental caries is an endogenous infection that results from microbiome dysbiosis with the involvement of acidogenic and aciduric species, which include low-pH non-*mutans streptococci*, *mutans streptococci* (MS), and numerous *Actinomyces* species that obtain a selective ecological advantage over other species^1^. Modern paradigms of dental caries aetiology concentrate on the major ecological pressure and micro-biome of the dental plaque that can modulate this to cause disease. The essential role that a dental plaque micro-biome symbiosis performs in preventing caries and enhancing oral health is also being recognized. Based on those principles, numerous ecological preventive strategies were advanced that could probably grow the arsenal of currently available caries-preventive measures^2^. Chlorhexidine seems like the gold standard antiplaque agent because of its substantive effects and excessive antimicrobial activity^3^. Regrettably, prolonged exposure to therapeutic agents could predispose to side effects, primarily the imbalance of the oral environment triggered by their bactericidal properties. It is due to this that antiplaque agents with very little direct bactericidal activity are sought^4^.

Several strategies have been proposed to rebalance the cariogenic plaque microbiome dysbiosis based on probiotics using different *Lactobacilli* species, including *L. rhamnosus* GG, *L. casei*, *L. reuteri,* and *Bifidobacterium* spp., which mediate the activity of odontopathogenic bacteria and hamper the growth of pathogens through the production of several antimicrobial agents^5,6^. A modern study confirmed that short-term exposure to fluoride from oral hygiene measures could not sustain anti-acid production activity, with the biofilms regaining acidogenicity over time without heeding the fluoride concentration^7^, fluoride therapy alone is not adequate for high caries challenges^8,9^. In this regard, thinking about a biomimetic method using selective natural agents with high intra-oral retention, and remineralizing without significant side effects may be a novel approach. Studies have shown that grape seed extract (GSE) could enhance the remineralization of carious lesions and appear to be distinct from the action of fluoride^10,11^. Furthermore, gallic acid, a primary constituent of GSE, enables mineral deposition^9^. Fortunately, grape seed proanthocyanin extract is considered a prebiotic agent due to its selective properties in stimulating of the probiotic microbiota and inhibiting the growth and activity of pathogenic bacteria^12^. Thus, probiotic-GSE aggregates can be referred to as ‘symbiotic’ as it alludes to synergism wherein the prebiotic compound selectively favours the probiotic compound^13^.

It has been identified that jelly candy is a great vehicle for transporting this latter symbiotic compound to the mouth because it is assumed that in this way the probiotic, grape seed extract, could be exposed for prolonged time to the tooth surface and within the oral cavity for beneficial effects. Further, the chewing impact itself will boost salivary stimulation and enhance the bicarbonate buffering system of the saliva^13^. Based on these considerations, the goal of our study is firstly to laboratory analyse the antibiofilm effects of probiotic grape seed jelly candy on the viability of *Streptococcus mutans* and *Actinomyces viscosus* colonization on enamel tooth surfaces, then visualise the morphological aspects using both scanning and transmission electron microscopy. Secondly, it is to quantitatively analyse the microhardness, energy-dispersive X-ray spectroscopy (EDAX), as well as the average fluorescence at both the demineralized and the remineralized zones of the laboratory carious lesions subjected to this jelly candy under a confocal laser scanning microscope.

Here, we hypothesize that an experimental symbiotic jelly candy could modify biofilms and diminish the cariogenic bacterial challenge, to favour the growth and dominance of healthy bacteria in the dental plaque microbiome.

## Materials and methods

### Sample preparation

**Figure 1:**
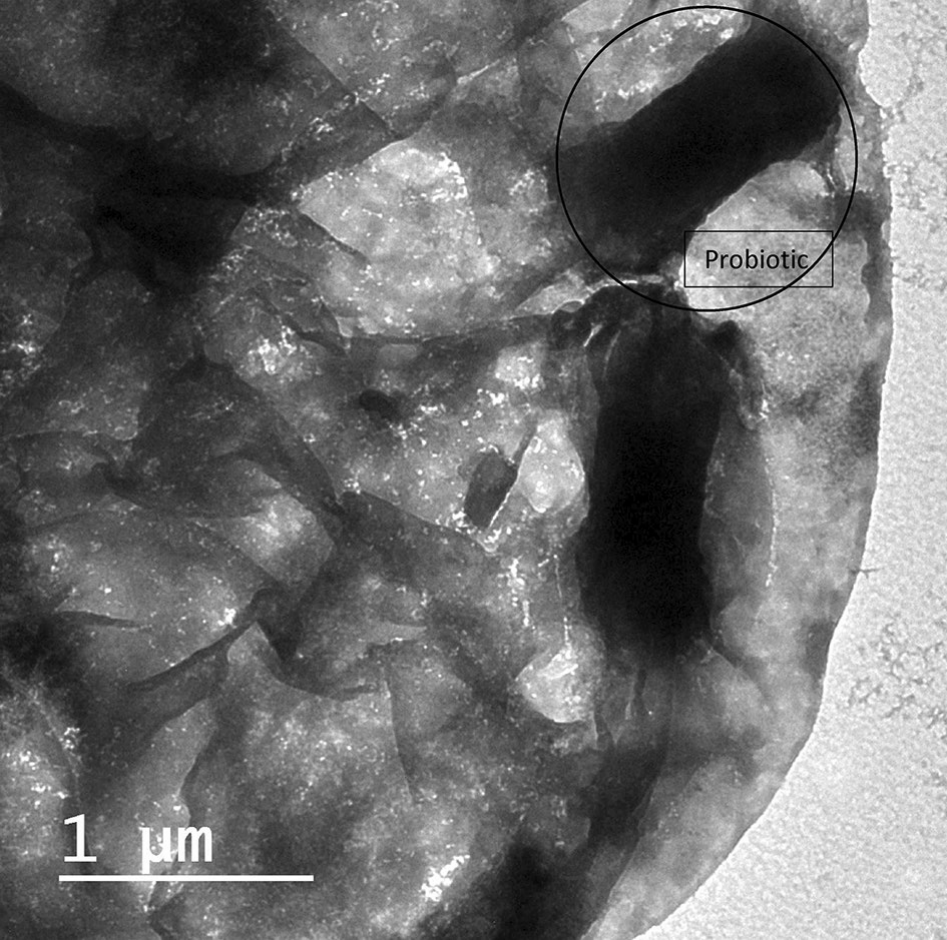
Transmission electron microscopy (TEM) showing part of a piece of jelly candy with probiotic bacteria (black circle) inside.

*Vitis vinifera* (Syrah grape) red grape seeds were received as production sachets from the manufacturer Ganklees factory (Alexandria Governorate, Egypt). The extraction of red grape seeds has been prepared at Packaging Materials Department, Chemical Industrial Research Institute (National Research Centre, Egypt). The extraction method used in this study was similar to the method explained by El-Sayed et al.^14^. 200 g of grape seed powder using a weighing balance (Pyrometro, Malaysia) was extracted with 800 ml of ethanolic solvent in a flask (ethanol: water, 70:30 (v/v) under constant agitation in a rotary shaker (Certomat Model S II, Sartorius, Goettingen, Germany) at 200 rpm, 45 °C, for 2 h. This was later centrifuged (Eppendorf Model 5810 R, Hamburg, Germany) at 5000 rpm for 10 min and subsequently decanted. Following, the residue was re-extracted for 2 h, and the supernatants were evaporated using a rotary evaporating instrument (Buchi Rotavapor R-215, Flawil, Switzerland) at the set pressure of 175 mBar, temperature of 52 °C, and speed of 95 rpm to remove the solvent. The ethanolic extract of grape seed powder was collected and stored in a glass container under frozen conditions^14^. The selected probiotic strain *Lacticaseibacillus rhamnosus* (NRRL B-442) was provided from the Dairy Microbiology Laboratory (Food Industries and Nutrition Research Institute, National Research Centre, Egypt), and grew in MRS broth and was incubated for 48 h at 37 °C anaerobically. The bacterial cells were obtained after centrifugation at 5000 rpm for 15 min using a cooling centrifuge at 4 °C^14^. The obtained cells were washed by saline solution (0.9% NaCl) and centrifuged at the same conditions. GSE Nano emulsion of 3% concentration with obtained probiotic cells in count around 10^7^ CFU/ml incorporated in a prepared jelly candy base formula (under patent registration no. 2021/976; Ministry of Scientific Research, Academy of Scientific Research and Technology, Egypt). GSE Nanoemulsion of 3% concentration with obtained probiotic cells in count around 10^7^ CFU/ml incorporated in a prepared jelly candy base formula. Firstly, probiotics jelly candy was prepared by heating water with honey and mixing well in a brass pan. After that, add the gelation solution by (melting the gelatin in warm water at 70 °C) to the previously prepared syrup and mix it well. Oleic acid, present in Tween^®^ 80, was added when the temperature reached 90 °C. The temperature of the mixture decreased to 40 °C before adding the previously developed formula that was designated by probiotic cells of *L. rhamnosus* with 3% GSE nanoemulsion^14^. Morphological study of the probiotic jelly candy supplemented with nanoemulsion grape seed extract (GSE) was evaluated using transmission electron microscopy (TEM) (Fig. 1).

### Microbiological assessment

#### Saliva collection

Unstimulated saliva accumulated from healthy female adult volunteers (aged 18–25) who had not been under antibiotic therapy for a minimum of 6 months. The volunteers had avoided eating, drinking, and brushing their teeth for at least an hour and a half. Saliva samples were pooled in sterile tubes and centrifuged at 12,000 rpm for 30 min at 4 °C. Pasteurization of the supernatants was performed at 65 °C for 30 min, followed by centrifugation and dispersion of the supernatant into sterile 50-ml polypropylene tubes, lastly stored at − 20 °C^15,16^. The efficacy of pasteurization was estimated by incubating 100 µl of processed saliva samples onto Columbia Blood agar plates, and the absence of CFU (i.e., the detection limit of 10 CFU/ml) was determined after 72 h at 37 °C on either aerobically or anaerobically incubated plates.

#### Enamel discs preparation

A total of one hundred and forty human teeth discs were selected for microbiological and remineralization testing methods. A total of one hundred and forty human teeth discs were selected for microbiological and remineralization testing methods. Enamel discs of 5 mm diameter and 2 mm thickness were obtained from extracted human posterior teeth (extracted for non-caries-associated orthodontic and therapeutic reasons). A cylindrical diamond-coated drill (trephine) was used perpendicular to the buccal surface of currently extracted teeth, and enamel surfaces had been wet ground with 600/800/1200/1500/2000-grit silicon carbide papers, respectively^17^. Ninety discs for microbiological evaluation have been indicated for counting bacteria viability, screening biofilm colonization via scanning electron microscopy (SEM), and transmission electron microscopy (TEM). These discs were protected with nail varnish on their sides and bottom, leaving most effective the buccal enamel surface exposed and fixed with a holder prepared with orthodontic wire and stored in a vertical position throughout the experimental procedures.

#### Bacterial strains and growth conditions

The microorganisms examined in this study were *Streptococcus mutans* (ATCC 25175) and *Actinomyces viscosus* (ATCC 19246). All strains had been received from MIRCEN (Microbiological Resources Centre, Cairo, Egypt) and were cryo-preserved at − 80 °C. Earlier than every experiment, two subcultures were prepared in tryptic soy broth (Difco, Sparks, MD, USA) for *Actinomyces* and *Streptococcus*. For *Lacticaseibacillus rhamnosus* (NRRL B-442), MRS medium (Merck, Darmstadt, Germany) was used, and all have been incubated at 37 °C for 24 h^4^.

#### Growing biofilm

The bacterial oral biofilm version system was assayed according to the version developed by Guggenheim et al.^18^ with a few adjustments as formerly described^19^. To allow for the formation of a salivary pellicle, ninety previously prepared enamel discs were randomly located in a sterile 96-wells polystyrene cell culture plate (Nunc A/S, Roskilde, Denmark), incubated with pasteurized saliva (800 µl) for 4 h, gently shaken (Eberbach's Microplate Vortex Laboratory Shaker E6120, USA), and kept at room temperature^18^. Following, saliva was aspirated from each well and replaced with a biofilm growth medium containing a mixture of 800 µl of pasteurized saliva, 21 amino acids (in total 2.6 g/l), vitamins, nucleotides, inorganic salts, trace elements, 10 mM glucose, and 200 µl of bacterial inoculum. In addition, the previous medium supported the growth of *Actinomyces* and *Streptococcus* species was supplemented with 800 µl of fluid universal medium (FUM), contained 67 mmol/l Sorensen’s buffer, pH 7.2, and was enriched with 0.15% sucrose and 0.15% glucose^18^. For the bacterial inoculum preparation, combining 1 ml of overnight precultures (OD550nm = 1.0 ± 0.02) of each of the two strains (*S. mutans*,* A. viscosus*) was performed.

The inoculum contained reproducibly between 10^7^ and 10^8^ CFU of each species per ml (mixed cultures of *A. viscosus* and *S. mutans* were obtained by mixing equal volumes of pure cultures). The biofilm growth under facultative anaerobic condition was obtained by mixing (95% N_2_ + 5% CO_2_) with (75% N_2_ + 5% CO_2_ + 20% O_2_) (Anaerobic Chamber (Anaerobic Workstation), ICB-AN3P, Bioevopeak, China) for up to 10 days at 37 °C^20^. Ninety enamel discs were randomly divided into three main groups (n = 30) according to the treatment agent exposed to the biofilm control group (G1) without treatment, the sterile saline group (G2), and the experimental group (G3). For the experimental group, the enamel discs immersed for 10 min twice/daily in 2 ml solution of 50 µg probiotic-GSE Jelly candy with distilled water^21^. The discs had been dip-washed for 10 s in sterile saline after which changed in their wells. Among every exposition, the plate incubated anaerobically at 37 °C, and the incubation medium changed daily in all experiments. Preceding medium replacement, the plates had been gently shaken, the discs were dip washed for 10 s in sterile saline, and transferred to a new plate wherein the fresh medium become delivered. After final exposure to the treatment occurred on the twenty-first day, the thirty enamel discs of each group have been stored in microtubes, each microtube containing ten samples were incubated for 48 h at 37 °C in an atmosphere of 95% N_2_ + 5% CO_2_ until use for testing procedures.

### Biofilm collection and analysis

#### Cultivation-based method (viable counting)

The ten enamel discs of each group had been washed with physiological saline and positioned in a sterile plastic Petri dish, and their surface was scraped with a sterile dental root curette. Then, the surface of the scraped disc and the Petri plate were rinsed with 1 ml of physiological saline. Aliquots of harvested biofilm were divided into diluted and spiral-plated onto the selective media for each strain: Mitis Salivarius agar + tellurite (Difco, Sparks, MD, USA) for *S. mutans*, and Trypticase Soy agar (Difco) for *A. viscosus*, all in 48–72 h of incubation at 37 °C. The CFU were performed for the biofilm-growing group (G1; BG), where biofilms were initiated from saliva and grown up, the probiotic-GSE Jelly Candy treatment group (G2; TG), and the sterile saline control group (G3; CG). The CFU per population for triplicate discs was averaged and subjected to a logarithmic transformation.

### Non-cultivation-based methods

#### Scanning electron microscopy (SEM)

The ten selected samples from each group were examined using scanning electron microscopy (High-Resolution Quanta FEG 250-SEM, Czech Republic). Samples were fixed for 24 h in a 2.5% glutaraldehyde solution, rinsed with 0.1 M Na-acetate buffer, and dehydrated with a graded ethanol series. Finally, they were dried, subjected to a critical point drier (Baltec, Leica Microsystems, Milton Keynes, UK) with CO_2_, and sputter coated with gold of 15 nm thickness using a Polaron SEM Coating Unit^22^.

#### Transmission electron microscopy (TEM)

For TEM, the last ten specimens for each group were immersed for 30 min in a fixative containing 2.5% glutaraldehyde and 1.5% paraformaldehyde in 0.1 M cacodylate buffer (pH 7.4). After washing threefold with 0.1 M cacodylate buffer and post-fixating with osmium tetroxide for 1 h, dehydration in an ascending ethanol series with post-staining in uranyl acetate was performed. Subsequently, all specimens of both groups were mounted for transverse sectioning of the adherent biofilms using a Leica Ultracut E (Leica, Wetzlar, Germany). In each ultrathin sections (60 nm thickness) mounted on filmed Cu grids and post-stained with lead citrate, the full length of the biofilm was studied with respect to its ultrastructure in a TEM (EM 900, Zeiss, Oberkochen, Germany) at 80 kV and magnifications of 3000 × to 20,000 ×^23,24^.

### Remineralization measurements

#### Artificial smooth-surface enamel lesion formation (demineralization procedure)

Fifty enamel discs that were used for remineralization measurements at three stages (sound, after demineralization, and after pH cycling) were covered with nail varnish leaving a window of 3 × 3 mm on the buccal surface. For the Vickers microhardness tester, twenty discs were tested, while the same ten discs were used for SEM, energy-dispersive X-ray spectroscopy (EDEX) (measured elements: Ca, P, F, C, Mg, N), and atomic force microscopy (AFM). For the confocal laser scanning microscope (CLSM), twenty discs were used for the linear depth of the induced remineralized area. On the sound stage, discs were randomly selected and measured for baseline values according to each test^25^. After measuring the sound enamel discs, enamel discs were kept in approximately 40 ml of demineralization solution (0.1 M lactic acid, 4.1 mM Ca (CaCl_2_ × 2H_2_O), 8.0 mM PO_4_ (KH_2_PO_4_) per enamel disc at pH 4.6 for 96 h in a sterile glass beaker at 37 °C^26^. In addition, 0.2% w/v Carbopol 907 (C907; BF Goodrich, Cleveland, OH, USA), a synthetic high molecular weight polymer, was delivered to the demineralization solution as a surface protective agent to maintain the enamel tooth surface and assist in creating subsurface lesions^27^.

### Post-treatment analysis of created incipient enamel carious discs

#### Testing of surface hardness

A total of 20 enamel discs were embedded on an acrylic resin block using for analyzing surface hardness through a Vickers microhardness tester (Shimadzu HMV-M Micro-hardness Tester; Newage Testing Instruments Inc., Southampton, PA, USA) under a 200 g load and a 15 s dwell time^28,29^. The mean values of the measured Vickers hardness number (VHN) of three indentations were obtained at baseline before enamel treatment, and after demineralization^30^. Then the enamel discs received remineralization treatment through exposure to a solution containing 5 g of probiotic-GSE nanoemulsion-based jelly candy dissolved in 2 ml of saliva (previously prepared during microbiological assessment) twice a day for 5 min in 15 days^31^. After each exposure, specimens were washed with distilled water for 20 s and then immersed in the saliva solution with a pH of 7.1 at room temperature until the next stage of the test. The saliva solution was replenished every 24 h^32^. In the meantime, the treated enamel tooth specimens were examined for surface hardness on day 7 and at the end of day 15. Outcomes expressed as the mean and standard deviation of VHN values at baseline, demineralization, and remineralization on day 7 and day 15^33,34^.

#### SEM, EDAX and surface topography assessment

The morphological analysis of ten specimens was performed using a SEM (Quanta 250 Field Emission Gun) attached to an EDAX unit (Energy Dispersive X-ray Analyses) and operating at an accelerating voltage of 30 kV. The enamel discs were first sputter-covered with gold in a vacuum evaporator (MED 010; Balzers, Balzers, Liechtenstein), after which they were microscopically analyzed to gain photomicrographs of the surface morphology of the treated specimens at 1000 × and 2000 × magnification. Images were obtained at the start of the experiment, at the demineralization stage, and after 14 days of remineralization treatment using probiotic-GSE nanoemulsion-based jelly candy^31^. The scanning area by SEM from the same sample was subsequently examined by atomic force microscopy (AFM) (Easyscan2 Flex) to evaluate surface topography. The EDAX point analysis (80 mm^2^, SDD [silicon drift detector], was performed to determine a qualitative elemental analysis of the same specimens (measured elements: Ca, P, C, Mg, and O). Five points per sample were randomly selected (300 μm^2^ per point), and the mean values were calculated^35^.

### Confocal laser scanning microscope (CLSM)

Following the creation of an artificial smooth surface enamel lesion (demineralization procedure) in the previously prepared enamel discs, the lower half of the 3 × 3 mm window of randomly selected twenty enamel discs was covered with nail varnish to serve as a control^20,25^. The treatment period of these enamel disc lesions was performed using probiotic-GSE jelly candy dissolved twice a day for 5 min as previously cited^25,31^. Subsequent to the remineralization treatment, twenty enamel discs were stained for 1 h with freshly prepared 0.1 mM Rhodamine B (C.I.45170) that was purchased from Sigma-Aldrich^®^ (Steinheim, Germany). The stained samples had been washed very well with phosphate buffer solution until there was no dye leaching out of the sample. All samples were installed on frosted glass slides with 80% glycerol, and the edges of the coverslip were covered^25^. The images with CLSM (CLS Leica TCS SL inverted microscope M, Leica Microsystems, Wetzlar, Germany) were captured from the buccal surface. Each image from either side of the mid-point (pre-treatment and post-treatment records) was measured from the occluso-cervical length of the tooth at 10 × magnification, and an argon laser was used at 488 nm wavelength for excitation and an emission range of 498–514 nm wavelength^45^. The buccal surface areas were scanned in microns (μm^2^) and the captured images (the Leica TCS SL in-built software, Germany) were calibrated for linear depth of fluorescence for the lower half of the 3 × 3 mm window demineralized (control) as well as the upper half remineralized (treatment agent) of each carious lesion^36^.

### Statistical analysis

The data were explored for normality using Kolmogorov–Smirnov and Shapiro–Wilk tests. The data showed a parametric (normal) distribution. For parametric data, a one-way ANOVA followed by Tukey post hoc test was used for comparison among the groups in non-related samples. A repeated measure ANOVA and paired sample *t*-test were used for comparison among the related samples. The significance level was set at P ≤ 0.05. A statistical analysis was performed with IBM^®^ SPSS^®^ Statistics Version 20 for Windows.

### Ethics approval and consent to participate

All performed procedures of this study carried out in accordance with relevant ethical guidelines and regulations of Helsinki Declaration. The Medical Research Ethics Committee (MREC), National Research Centre of Egypt (33 El Bohouth St., Dokki, Giza, Egypt) with reference number “19336/2022”, approved all experimental protocols. For the collection of isolated teeth, informed consent was obtained from all participants.

Experimental research and field studies on plants including the collection of plant material are comply with relevant guidelines and regulation.

## Results and discussion

### Results

Transmission electron microscopy (TEM) image analysis in (Fig. 1), showing a bacteria cell *Lacticaseibacillus rhamnosus* (NRRL B-442) (black circle) covered by a smooth and homogeneous surface part of a piece of jelly candy.

**Figure 2:**
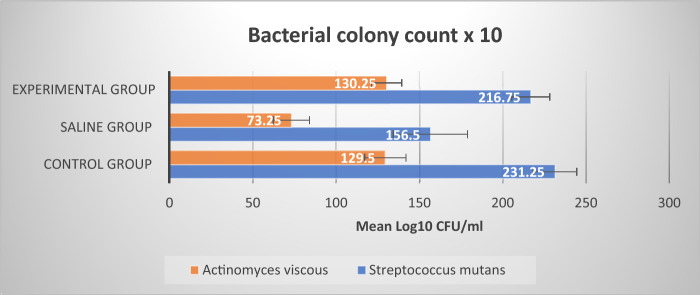
A bar graph representing *S. mutans* and *A. viscosus* colony counts for 21 days biofilm control group without treatment (G1), and after exposure to sterile saline group (G2) and probiotic-GSE Jelly candy experimental group (G3). The highest mean value of the reduction in *S. mutans* colony count was found in experimental group (156.50 ± 22.34-log10 CFU/ml) (*P* ≤ 0.001), while no statistically significant difference was found between Control group (G1) and sterile saline group (G2) in mean value of *A. viscosus* and *S. mutans* bacterial counts, where (*P* = 0.957) and (*P* = 0.318) respectively.

In the biofilm assays (Fig. 2), the biofilm control group (G1) contained (129.50 ± 12.37 log_10_ CFU/ml) of *A. viscosus* and (231.25 ± 13.15 log_10_ CFU/ml) of *S. mutans* after 21 days biofilms were initiated from saliva and grown up without treatment (G1), as well, (130.25 ± 9.03 log_10_ CFU/ml) of *A. viscosus* and (216.75 ± 11.35 log_10_ CFU/ml) of *S. mutans* after exposure to sterile saline (G2), while no statistically significant difference was found between Control group (G1) and sterile saline group (G2) in the mean value of *A. viscosus* and *S. mutans* bacterial counts, where (*P* = 0.957) and (*P* = 0.318) respectively. After 21 days treating with 50 µg/2 ml probiotic-GSE nanoemulsion-based jelly candy experimental group (G3), bacterial counts for *A. viscosus* were reduced to (73.25 ± 10.90 log_10_ CFU/ml) (*P* ≤ 0.001) in comparison to control group (G1). An experimental group was also the most inhibitory in *S. mutans* colony formation, reducing biofilm development to 74.8 log_10_ (156.50 ± 22.34 log_10_ CFU/ml) (*P* ≤ 0.001). Accordingly, a single 10 min treatment daily for 21 days with jelly candy resulted in a 68% decrease in *S. mutans* colony formation, reducing biofilm development.

**Figure 3:**
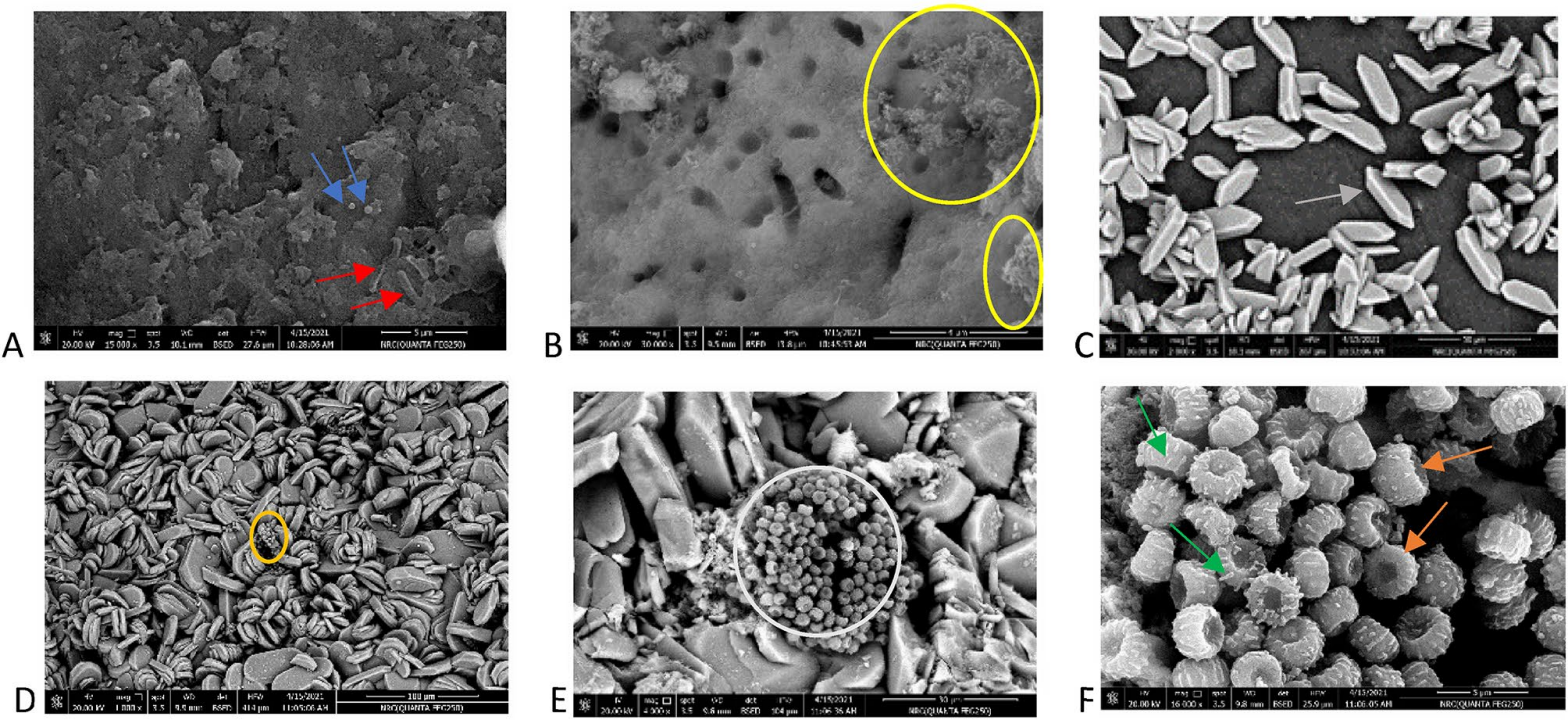
SEM images of enamel discs showing: (**A**) 21 days growth of *S. mutans* (blue arrow) and *A. viscosus* (red arrow) without treatment. (**B**) biofilm growth (yellow circle) appeared after 21 days exposure to sterile saline for 10 min twice/day. (**C**) SEM image of the crystals (grey arrow) formed on the enamel surface after using probiotic-GSE nano emulsion-based Jelly candy and (**D**) was displayed covering of bacteria cells by Jelly candy (orange circle). In (**E**,**F**), it showed after magnified micrograph of (**D**) (orange circle) at magnification (× 4000) (**E**) presence of colony of cocci like bacteria (white circle). Magnification of (**D**) (orange circle) at (× 16,000) (**F**), it was clearly seen covering of *S. mutans* (dark orange arrows) by Jelly candy with the attachment of bacilli like bacteria (*L. rhamnosus*) (green arrows) on its surface.

**Figure 4:**
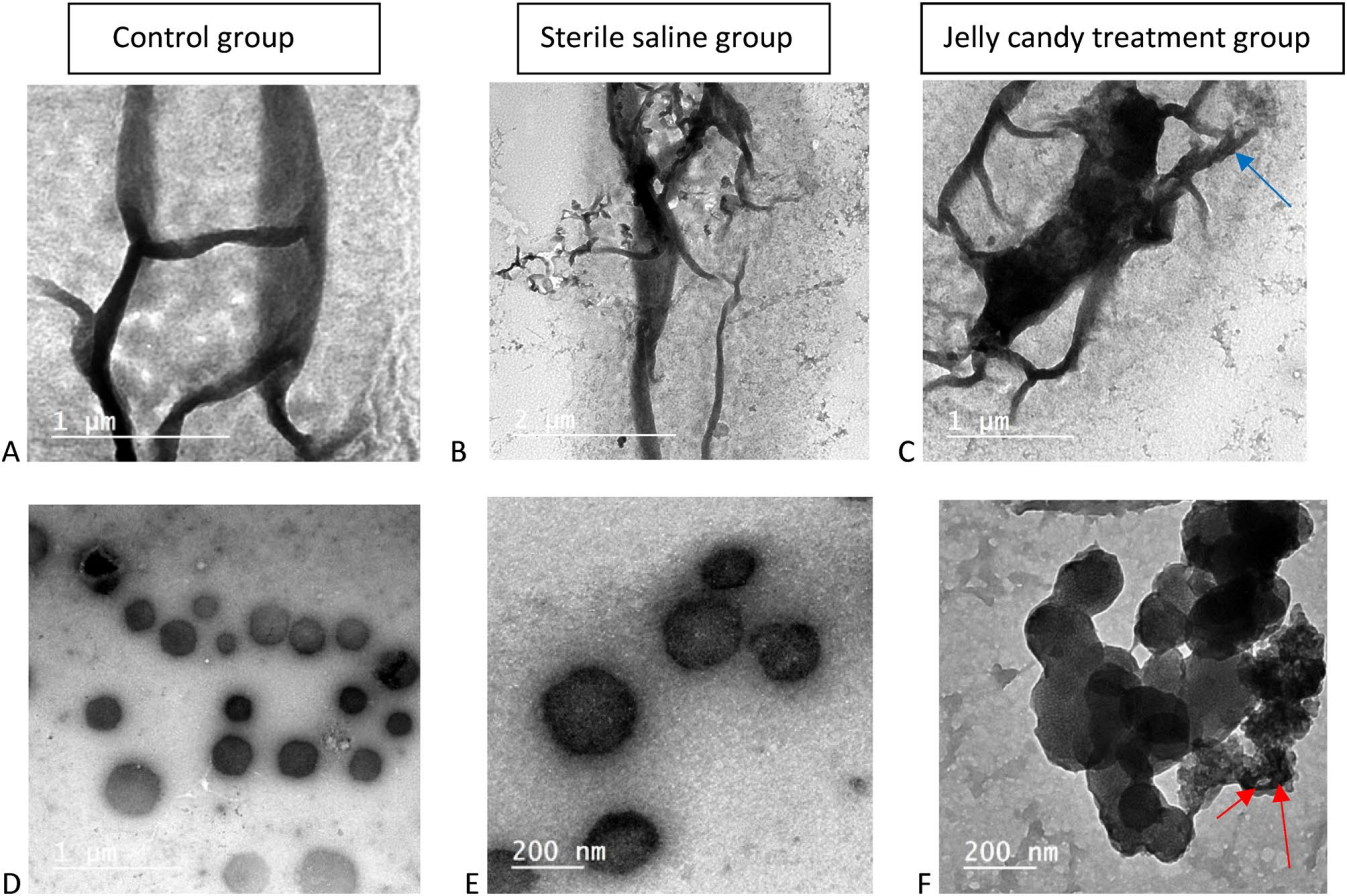
TEM images show a significant modification on *Streptococcus mutans cells* (**F**) (red arrows) with a minor change on *Actinomyces viscosus* outside of cells appeared (**C**) (blue arrow) after 21 days exposure to probiotic-GSE nano emulsion-based Jelly candy. In case of biofilm growing without treatment (**A**,**D**) and after exposure to sterile saline (**B**,**E**), rarely, changes to the cells surfaces are visible.

The SEM image of enamel discs after 21 days without treatment shows (Fig. 3A) the growth of *Streptococcus mutans* (*S. mutans*) and *Actinomyces viscosus* (*A. viscosus*), while (Fig. 3B) the SEM image obtained after 21 days exposure to sterile saline shows *S. mutans* could be appeared more dominant to a certain degree than *Actino. viscosus*. The SEM images suggest newly formed crystals on the enamel surface after using probiotic-GSE nano emulsion-based jelly candy (Fig. 3C) and trapping *S. mutans* were displayed in jelly candy (Fig. 3D), as well as clearly visualized (Fig. 3E,F) at magnifications (4000 ×), (16,000 ×) respectively. The TEM images show a significantly altered morphological structure of *S. mutans* (Fig. 4F) compared with the control (Fig. 4D) and sterile saline group (Fig. 4E). In case of *A. Viscous*, a minor change on the outside of cells appeared (Fig. 4C) after 21 days of exposure to jelly candy, with rarely visible changes to the cell surface (Fig. 4A,B) of the control and sterile saline groups, respectively.

**Figure 5:**
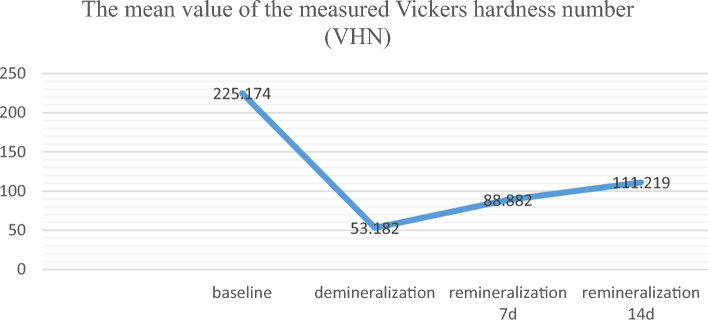
Line chart provides the data for the mean value of the measured Vickers hardness for the sound enamel discs at baseline, after demineralization, and after 7 and 14 days remineralization treatment with probiotic-GSE nano emulsion-based Jelly candy. VHN was significantly different between stages, and treatment times (*P* < 0.0001). During the period of treatment from day 7 to 14, there was highly statistically significant increase in mean value of VHN at *P* < 0.01.

**Figure 6:**
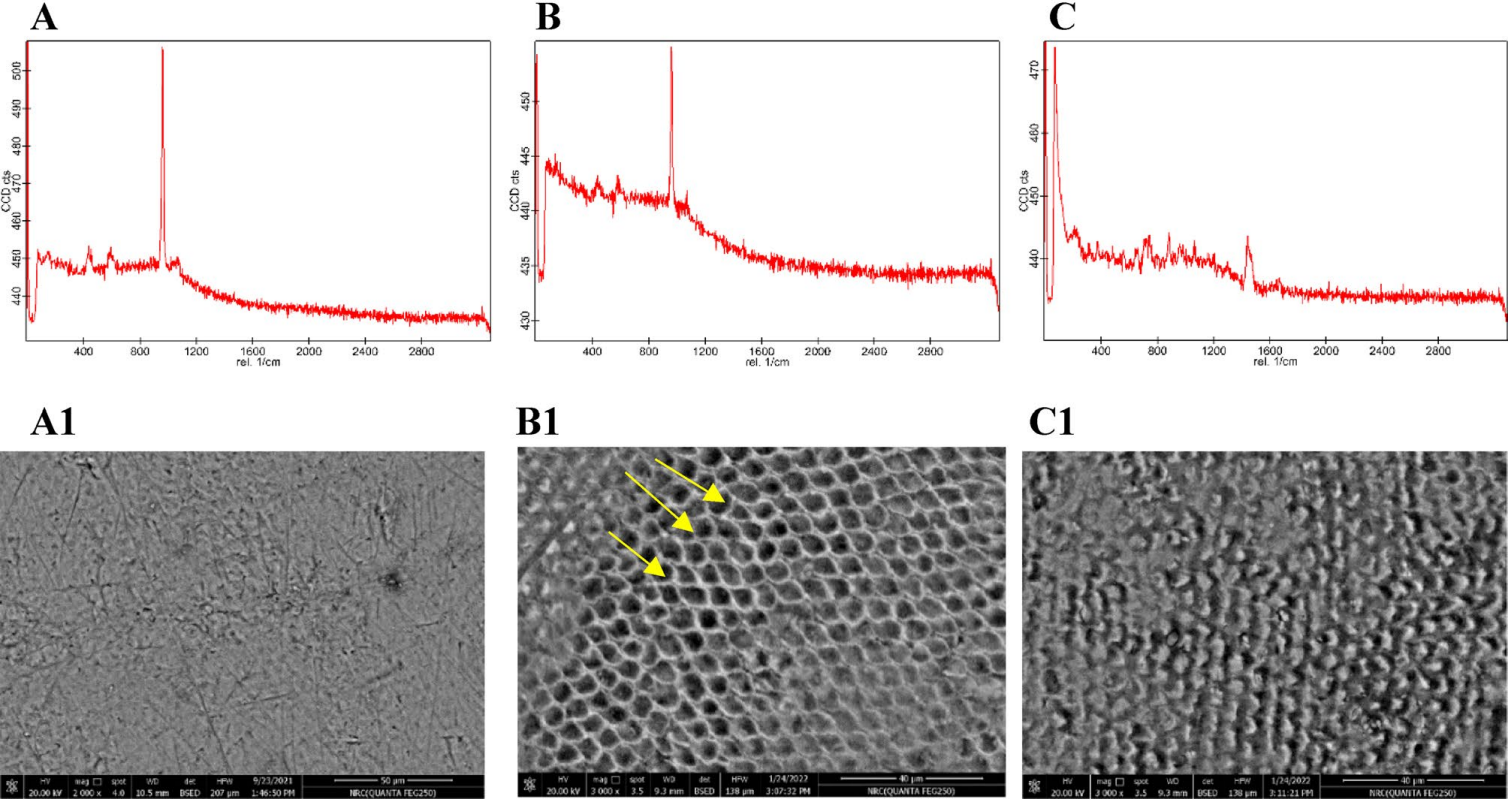
(**A**–**C**) line profiles of AFM for the surface enamel discs representing SEM images of (**A1**,**B1**,**C1**) respectively showing the capability of AFM to evaluate surface topography Corresponding SEM images of the same sample. Data obtained before the start of the experiment (**A**,**A1**), at demineralization stage (**B**,**B1**) showing the hexagonal structures denoted by the yellow arrows related to the exposure of enamel rods, and after 14 days demineralization treatment with probiotic-GSE Jelly candy (**C**,**C1**).

In the remineralization analysis, Fig. 5 provides the data for VHN values for the same samples at baseline, before demineralization, and after 7- and 15-day exposure to probiotic-GSE nanoemulsion-based jelly candy. VHN was significantly different between stages, and treatment times (*P* < 0.0001). VHN values decreased considerably following demineralization, as shown in the line chart in Fig. 5, with a statistically significant gradual increase in VHN values at days 7 and 15 of remineralization. The findings from AFM in Fig. 6 have revealed that the topography structure scanned by AFM probe could be related to the dynamic changes of the enamel surface of the same sample. The obtained results in (Fig. 6B) indicated that the backscattered intensity on the tooth surface became stronger after demineralization and gradually decreased following 14 days of remineralization treatment with probiotic-GSE nano emulsion-based jelly candy, as shown in (Fig. 6C). To validate the AFM results presented in (Fig. 6A–C) the differences between the healthy, demineralized, and remineralized teeth were examined by SEM. Figure 6A1,C1 shows the SEM images of the healthy and remineralized enamel surfaces obtained at 2000 × and 3000 × magnifications, respectively.

**Figure 7:**
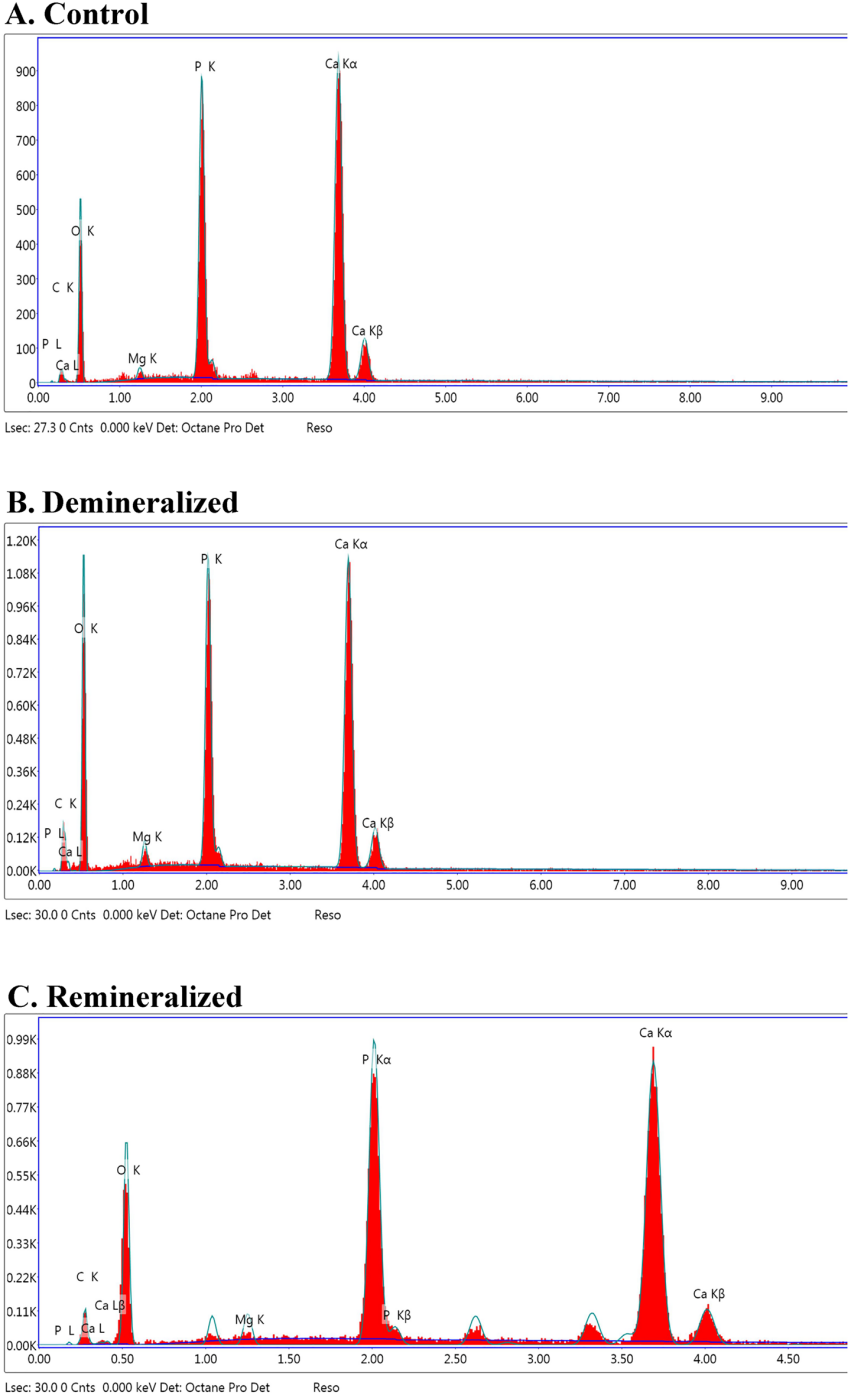
Energy-dispersive X-ray spectra collected on sound enamel, demineralized, and after remineralized lesion were compared. The X-ray spectra were collected with an accelerating voltage of 15 kV.

In Fig. 6B1, display the SEM images of the demineralized tooth obtained at 3000 × magnifications. In contrast to the results presented in (Fig. 6A1,C1), the enamel surface after etching contains the hexagonal structures denoted by the yellow arrows in Fig. 6B1, which could be related to the exposure of enamel rods. The observations of the inorganic compounds on the enamel surface done by means of EDX in the form of graphs are shown in (Fig. 7A–C) and corresponding entries in Table 1A–C for the weight % of these compounds (Ca, P, C, O, and Mg) in surface enamel samples at three stages (sound, after demineralization, and after 14 days of remineralization treatment with probiotic-GSE nanoemulsion-based jelly candy). The surface weight % of Ca, P, C, O, and Mg were significantly affected by demineralization time and treatment, as shown in (Fig. 7B and Table 1B) also demonstrate a detectable drop in wt% of both Ca (15.36 weight%), and P (11.12 weight%) following the demineralization stage in comparison to sound stage Ca (23.38 weight%), and P (15.38 weight%). The surface weight % of Ca was significantly affected by treatment, as specimens received a high surface calcium weight % (29.75 weight%) irrespective of demineralization stage, as shown in (Fig. 7C and Table 1C).

**Table 1:** The results mean weight% of chemical elements at the enamel surface at different stages (A, sound; B, following demineralization; C, after treatment) (error bars displayed for better clarity).

Element	Weight %	Atomic %	Net Int	Error %
(A)				
C K	3.83	6.43	2.78	24.35
O K	56.2	70.81	79.45	11.42
Mg K	1.2	1	8.66	18.86
P K	15.38	10.01	244.16	4.83
Ca K	23.38	11.76	352.66	2.49
(B)				
C K	12.41	18.59	17.04	13.49
O K	59.33	66.74	156.88	10.49
Mg K	1.77	1.31	21.19	13.38
P K	11.12	6.46	288.97	4.62
Ca K	15.36	6.9	388.06	2.13
(C)				
C K	9.29	15.95	12.35	14.52
O K	44.97	57.98	89.36	11.46
Mg K	0.57	0.49	6.3	24.07
P K	15.42	10.27	236.63	3.78
Ca K	29.75	15.31	311.33	2.58

**Figure 8:**
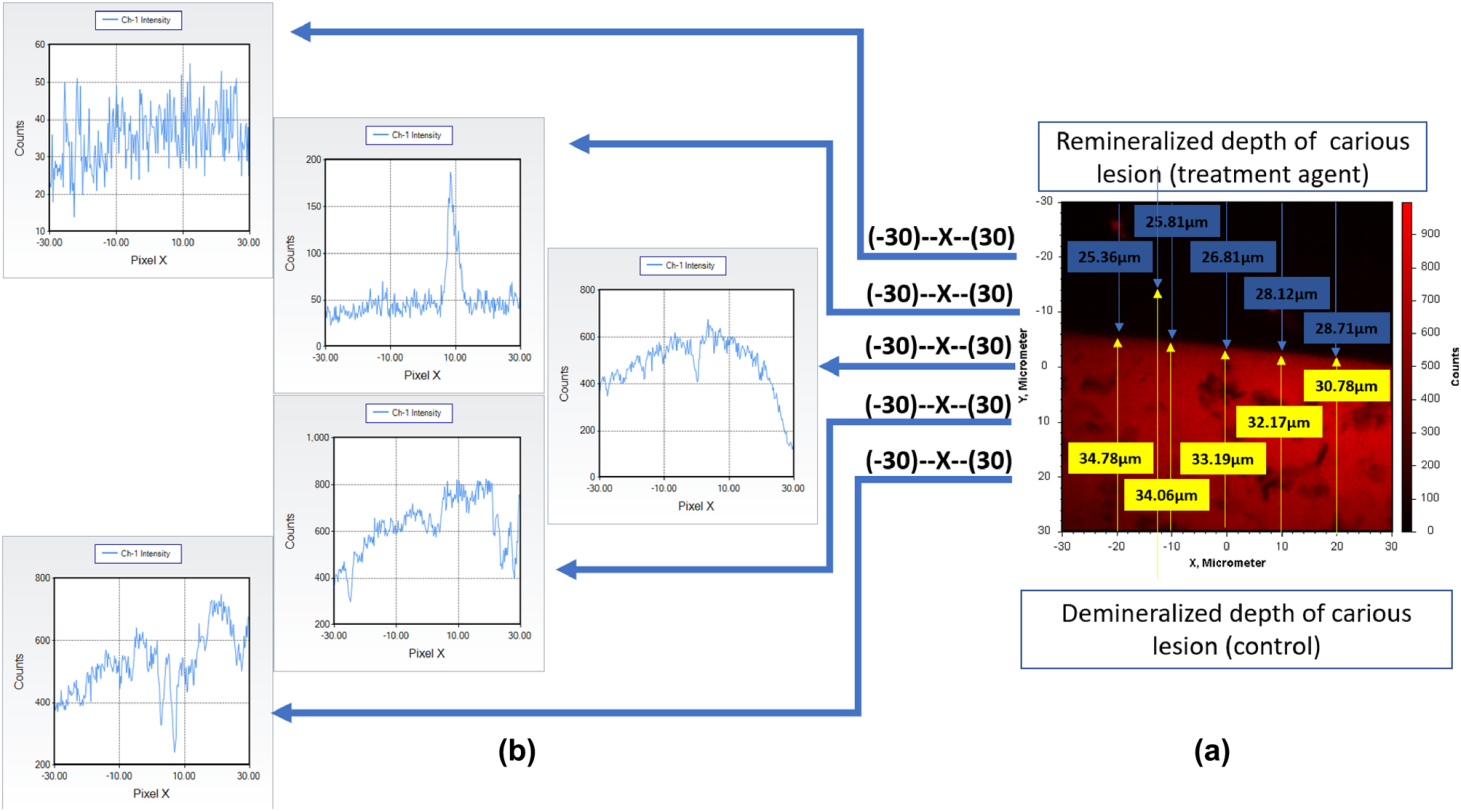
(**a**) Representing the linear depth of lesion in μm as seen through confocal laser scanning microscope and (**b**) showing calibrated image for graphs of the intensity of fluorescence along both de mineralized and re mineralized areas.

EDX detected stronger carbon signals at the demineralization stage (12.41 weight%) in contrast to sound stage (C, 3.83 weight%), and gradually carbon weight% decreased in the mineralized layer formed at the enamel surface after treatment (C, 9.29 weight%). Also, the amounts of inorganic compounds oxygen, and magnesium content on the enamel surface were obviously increasing (O, 59.33 weight% and Mg, 1.77 weight%) following demineralization and markedly decreasing after treatment (O, 44.97 weight% and Mg, 0.57 weight%). The induced carious lesion depths were assessed under CLSM Fig. 8 by measuring from the enamel surface to the deeper zone of the lesion, and an average of ten measurements were taken from every image that was captured both in the demineralized and remineralized zones of the (3 × 3 mm) window of every specimen. The captured image shows the graph of the intensity of fluorescence was displayed in (Fig. 8b), while the image displays the length measurements (Fig. 8a). On comparison between demineralized, and remineralized teeth a significant remineralization, with a reduction in lesion depth was evident for the upper half of the remineralized zone of the window specimen following seven days treated with probiotic-GSE nanoemulsion-based jelly candy. Also, the extent of 0.01 mM Rhodamine B dye penetration was seen as fluorescence in the specimens under argon laser calibration and recorded with the in-built software [Leica TCS SL, Germany]. The findings show that when a 0.1 mM solution of rhodamine B dye was used, the lesion area correlated well with the mineral loss. However, the average lesion fluorescence best represented mineral loss, as shown in (Fig. 8a) and that the highest lesion depth value following demineralization was 34.06 ± 7.81 μm with an increase in the intensity of fluorescence as noticed in (Fig. 8b). In contrast, the highest lesion depth value of remineralization was 28.76 ± 3.46 μm corresponding to decrease in the intensity of fluorescence. Hence, the values were significant [*P* < 0.001] and the remineralization values were lower than the demineralizations that were obtained under CLSM (Fig. 8).

### Discussion

Dental caries arises from disturbance in the biomineralization of dental enamel. Demineralization is caused when the pH of the oral biofilm drops below 5.5 by an acidic attack through dietary acid consumed in food or drink and by a microbial attack from specific bacterial strains present in the oral biofilms on the enamel tooth surface^37^. Most synthetic sugars are not or slightly fermented via oral bacteria, so they no longer lower the pH^38^. Furthermore, chewing gum yields improved remineralization of enamel by increasing calcium and phosphate levels in the oral cavity through the enhanced volume of stimulated saliva during mastication, with concomitant ability to clear fermentable carbohydrates and acids on the teeth surfaces^39,40^.

A symbiotic relationship and diverse composition of the oral microbiome are the main keys to oral health, even though the exact composition of what is tentatively referred to as “the oral microbiome in health” is not always known. Elimination of specific pathogens could attribute to oral microbiome dysbiosis through a shift in microbiome composition to a less diverse route, and this dynamic presents opportunities for oral diseases^59^. In our microbiological assays, the probiotic-grape seed extract (GSE) nanoemulsion-based jelly candy might better combat an already formed cariogenic biofilm after 21 days, resulting in a decrease bacterial count for *A. viscosus* with a significant 68% decrease in *S. mutans* colony formation reducing biofilm development. Hence, our study supports the conclusion that the gradual decrease in specific bacterial pathogens from the oral cavity through chewing gum is most advantageous to surprising ecological shifts that could be alternate the relationship among the oral microbiome and the host, which encourage homeostasis and thus contribute to the oral microbiome at health^41^.

The use of probiotic lactic acid bacteria (LAB), specifically *Lactobacilli*, as a natural barrier against pathogens might be very efficient due to their production of antimicrobial metabolites^42^. However, it is important to offer this sort of antimicrobial product in a natural, close to the food form, which is the most suitable for consumers. According to WHO (World Health Organization, 2014)^43^, antibacterial resistance to bacterial pathogens is turning into a significant public health problem. In this respect, our study represented combinations of antimicrobial compounds (probiotic and grape seed extract (GSE) nanoemulsion) in jelly candy that could enhance the effectiveness of the antimicrobial product and permit a reduction in the dose of every compound. Similarly, synergism in antimicrobials could be applied to help delay the emergence of the resistance of specific bacterial pathogenic strains in vivo^44^.

A smooth and homogeneous surface of jelly candy under transmission electron microscopy (TEM) as revealed in (Fig. 1). The addition of oleic acid to the interior components of the developed formula of jelly candy could cause a change in its morphological surface. In addition, it is suggested that the availability of certain fatty acids in a developed formula is a factor that contributes to maintaining the probiotic bacterial cell wall morphology inside the jelly candy and the protection in contradiction of acidic conditions in the oral cavity^45^.

The morphological aspects of microbial colonization on natural tooth surfaces have been investigated using both scanning electron microscopic (SEM) and transmission electron microscopic (TEM) analyses. In the present study, the coating of *S. mutans* was clearly visualised in probiotic-GSE nanoemulsion-based jelly candy under SEM. Previously, observations showed that chewing gum participates in the renovation of oral health and dislodges loosely bound oral bacteria from oral surfaces^21,39^. Hence, our experimental chewing gum can inhibit pathogenic bacteria and eliminate them from the oral cavity. The TEM micrographs presented in the current study support the conclusion that a significantly altered morphological structure of *S. mutans*, with a minor change on outside of cells, appeared in *A. viscosus* after 21 days of exposure to jelly candy. However, prior to TEM evaluation, samples must go through large processing steps, and protein denaturation throughout fixation and dehydration of specimens may cause a condensing effect, thereby changing the ultrastructural morphological appearance of the bacterial surface will not be affected by these possibly occurring artifacts^23^. In the present study to support the microbiological conclusion, the antimicrobial activity of grape seed extract (GSE) was attributed to the availability of phenols observed in nanoemulsion^46–48^. Consistent with the preceding results, the primary active polyphenol compounds have been catechin, chlorogenic acid, and methyl gallate, which have antimicrobial action. Further, our results were in agreement with the previous study and showed that probiotics can strongly antagonise cariogenic species, such as *S. mutans*^49^. In addition, the acids produced by probiotic *lactobacilli* that have an immediate effect on the growth of *S. mutans* were investigated^21,50^.

The existing SEM images displayed newly formed crystals on the enamel surface after using probiotic-grape seed extract (GSE) nanoemulsion-based jelly candy. Xie et al. suggested that GSE ought to contribute to the deposition of minerals at the superficial layer of the lesion^51^. Previous work reported that a large concentration of calcium in grape seeds (55.74%) was obtained with a lower concentration of phosphorus (15.34%)^11^. Accordingly, these newly formed crystals could be calcium phosphate that exists in a soluble crystalline phase called brushite, contributing to enhancing the remineralization of enamel subsurface lesions^52,53^.

The current study displayed a strong correlation between the hardness of the enamel surface and its chemical content. Regions with a higher concentration of calcium and phosphorus showed the highest Vickers hardness values (VHN) after treatment with probiotic-GSE nanoemulsion-based jelly candy. However, regions with higher sodium and magnesium concentrations displayed an alternative trend following the demineralization stage. The present results have been demonstrated by several others^54,55^, who reported that the lower microhardness values were associated with a concurrent decrease in calcium and phosphorus contents. However, the obtained results from EDX showed a detectable drop in weight% of both Ca and P after demineralization that could affect the enamel ultrastructure by way of increasing interprismatic spaces and converting individual crystallites by their dissolution^56^. Moreover, remineralization of demineralized crystallites using jell candy is not uniform, with ion deposition into central defects and onto the crystal periphery, further growing the irregularity of the crystallites^56,57^. This creates regions of variable porosity with nonuniform sizes and spatial distributions.

In addition, the obtained results from AFM could be related to the dynamic changes of the enamel surface as the backscattered intensity on the tooth surface became stronger after demineralization and gradually decreased following a 14-day remineralization treatment with jelly candy. It was formerly shown that the acid caused demineralization induced the appearance of enamel rods at the etched enamel surface^58^. However, our study demonstrated the improved backscattered intensity at the enamel surface that is likely associated with the presence of these rods. Hence, these effects agree with the data obtained in a previous study.

The current study validated the AFM results through SEM. The SEM image results presented the enamel surface after etching, which contains hexagonal structures that could be related to the exposure of enamel rods. Previously, work supported the idea that these structures represent enamel rods or prisms, which include hexagonal hydroxyapatite crystals^58,59^. Therefore, the SEM images revealed that tooth demineralization caused the dissolution of superficial calcium hydroxyapatite, which exposed the enamel rods and enhanced the backscattered intensity from the tooth surface.

The induced carious lesion depths were investigated under CLSM by measuring from the enamel surface to the deeper zone of the lesion. The findings show that the highest lesion depth value of remineralization was 28.76 ± 3.46 μm corresponding to a decrease in the intensity of fluorescence. When a 0.1 mM solution of rhodamine B dye was used, the lesion area correlated well with the mineral loss^60,61^. As previously mentioned in the present study, calcium and phosphorus in grape seeds could exist in a soluble crystalline phase called brushite and enhance the remineralization of enamel subsurface lesions^52,53^. However, the average lesion fluorescence those best-represents mineral loss relies upon their speculation that rhodamine B penetrates the voids and pores created throughout tooth demineralization, which is a favourite approach of assessment over birefringence via CLSM^25^.

A variety of recent anti-cariogenic and remineralizing agents are available on the market. Therefore, it is vital to understand the advantages and efficacy of these products before recommending their use in clinical practise. The advantages of our study are the inclusion of probiotic-GSE nanoemulsion-based jelly candy over probiotic suspension, including the facility to apply it as a topical agent, the possibility of storage, and prolonged exposure of microbial cells inside the oral cavity. The clinical significance of probiotic Jelly candy Supplemented with nanoemulsion grape seed extract (GSE), its importance may continue to emerge as the importance of fluoride did in the past for remineralization potential and significant caries reduction. Therefore, the practical application of the developed formula, Probiotic-GSE Jelly candy, has a potential therapeutic value and may have a high cost-effectiveness in routine setting use. However, the results obtained in this study can serve as a guide for further clinical investigations.

## Conclusion

In summary, within the limitations of this experimental study, the nanoemulsion of grape seed extract and *Lacticaseibacillus rhamnosus* (NRRL B-442) probiotic strain impregnated in jelly candy were able to be both anti-cariogenic and remineralizing, suggesting possible use in tooth decay prevention and reduction. Furthermore, a long-term clinical study is recommended to determine its efficacy.

## Data Availability

The datasets used and/or analysed during the current study available from the corresponding author on reasonable request.
